# The use of photovoice to transform health science students into critical thinkers

**DOI:** 10.1186/s12909-021-02656-1

**Published:** 2021-04-23

**Authors:** Firoza Haffejee

**Affiliations:** Department of Basic Medical Sciences, Durban University of Technology, Durban, South Africa

**Keywords:** Co‐creation of knowledge, Photovoice, Public Health, South Africa, Transformative education

## Abstract

**Background:**

Students are often inadequately prepared for higher education, particularly concerning independent learning and critical thinking. These attributes are essential, especially in health science students as health care needs are complex. Innovative methods of teaching that promote these attributes are thus required. One such method, which has been included previously in other disciplines is photovoice, a participatory method, in which students become co-creators of knowledge. The aim of the study was to determine whether photovoice would promote critical thinking in students enrolled for a module in Public Health. The study also aimed to analyze the experiences of students using this methodology, as part of their learning.

**Methods:**

Photovoice was introduced to a class of 56 chiropractic and homeopathy students registered for a module on Epidemiology: Public Health in 2019. Students working in self-selected groups were required to take photographs of environmental factors, involved in causing disease. After engaging in a group dialogue, one photograph was selected for presentation in class, with a discussion of how environmental factors visible in the photograph affect the health of individuals. Presentations were assessed based on the picture, presentation quality and ability to answer questions. Focus group discussions were subsequently held to understand the experience of students with this new teaching method. Data was analyzed using thematic analysis.

**Results:**

Students established that it was a positive experience. They recognized the lived realities, within the community, that cause disease. The assignment demonstrated how learning can occur beyond the lecture room and extend into communities. Students offered realistic solutions to health problems that were confronted by communities. In addition, students participated in unintended community engagement.

**Conclusions:**

The incorporation of photovoice into undergraduate teaching in the health science module promoted higher order learning such as problem solving and critical-thinking. Students transformed from rote learners to critical thinkers who reflected upon what they were taught and how this related to the lived realities of the community. Student communication improved as they disseminated knowledge to others. Teaching using this alternative pedagogy has the potential to produce graduates who are responsive to the local needs of the community.

## Introduction

The predominant teaching method in a typical South African university is the didactic teaching mode of lecturing with an emphasis on information delivery. Printed and electronic versions of the lecture content, reinforce learning. In general, students are passive recipients of information within the teaching and learning space. A definitive result from such a modality of engagement prevents inculcation of critical thinking, as students become passive recipients of knowledge [1]. Hence,  there is a  need for curriculum transformation so that all students become critical thinkers, who will succeed both as graduates and future professionals [2].

Critical thinking involves problem solving, analysis and interpretation of information as well as decision making. It incorporates formulating questions and seeking answers to these questions [3]. Critical thinking occurs in response to a challenge, when conducting investigations and engaging in creative tasks [3]. In the health sciences, these skills will be of particular importance when deciding on the course of action to take in instances where patients present with atypical symptoms and the health care practitioner is required to think clearly and rationally about the course of action to take [4]. Allied health students may also become promoters of health care, thus requiring the skills that allow them to make clear judgements and be able to communicate confidently and effectively [5, 6].

The transformational teaching agenda commits critical engagement with students in the teaching & learning process. This fosters reflective thought, creative problem solving, and is learner-centred and interactive [7]. Within the health sciences, learning has to be relevant and responsive to community health needs in order to improve the social accountability of graduates [8]. Knowledge of disease and the social determinants of health, which refers to the conditions in which people live, grow and work; is also necessary [6, 9]. Venkatapuram [10] state that “the social environment interacts with the psychological functioning of the individual which then influences the biological functioning”. The Health Professions Council of South Africa (HPCSA) has reiterated the necessity for improvement through health promotion, educating communities and the development of reflective learning [9]. Health care needs in South Africa, as in other countries, are complex and require critical thinking as well as competencies and capabilities that address these needs. Competence is the ability to perform effectively, often in a familiar context. Capability goes beyond the achievement of competence in familiar situations to performing in an unfamiliar context with unfamiliar problems. Capability requires three attributes: The ability to learn for oneself, to be able to perform in a new situation, where learnt skills can be applied to a new environment, and to have powers of judgement [11].

Innovative methods of teaching that promote active learning and critical thinking are thus required [12]. Innovative methodologies of transformative education, including group projects, role plays and simulations, have previously been utilized to enhance learning of Public Health at South African universities [13–15]. These studies have demonstrated the benefits of inculcating critical thinking that facilitates independent scholarship. Hence, such a modality needs to be sustained to improve the teaching and learning environment.

Students become co-creators of knowledge within the learning landscape. This pedagogy engages students as active, participatory learners who develop into critical thinkers. This dialogical concept of learning is similar to the concepts of Paolo Freire who maintained that critical thinkers would contemplate different ways of living [16]. One such method that has previously been used in other disciplines such as education, social science and geography, is photovoice [17–19]. Photovoice, commenced as a participatory research method, in which the research is a cooperative process between the researchers and participants, both of whom are actively involved in the research process and hence in co-creating knowledge [20]. It was developed in China in the early 1990s, when rural women were requested to photograph their challenges on healthcare issues [21]. Photovoice empowers participants, who are often from socially marginalized groups and are unable to articulate their thoughts appropriately into words. Thus, the research process becomes an educational and inspiring process for both the researcher and the participants [22]. In South Africa, it has been employed as a research methodology in Public Health [23, 24]. However, within the teaching of Public Health to undergraduate university students this methodology has been minimal.

In South Africa, learners from basic education are ill prepared for higher education, in particular they are not equipped for independent learning nor critical thinking [25]. In response to this, the new strategy for the Durban University of Technology (DUT), Envision 2030, has four perspectives of “Stewardship, Systems & Processes, Sustainability and Society”. These are scaffolded with society at the top as this is the ultimate point of impact. Embedded among these perspectives are: “innovative curricula and research”, “a distinctive education” and “an engaged university” [26]. The university has recently included general education modules which focus on these attributes. These values, however, also need to be introduced and embedded within the entire curriculum. In committing to “innovative curricula”, photovoice was incorporated into the Public Health module that translates Envision 2030 into reality.

The research questions that guided the study were:

- Can photovoice be used effectively in public health education?
- What are the perceptions of students regarding photovoice as a method of learning?
- Can photovoice transform students into critical thinkers, capable of analyzing problems in various contexts?

The aim of the study was to determine whether photovoice would promote critical thinking in students enrolled for a module in Public Health. The study also aimed to analyze the experiences of students using this methodology, as part of their learning.

## Methods

This qualitative study was conducted with the sample cohort of second year health science chiropractic and homeopathy students registered for a module on Epidemiology: Public Health. The module comprises of basic epidemiological principles and incorporates aspects such as causes of communicable disease, types of research surveys, basic calculations, data presentation in epidemiology and control measures for various diseases. It also describes the interaction of pathogen, host and environmental factors in disease causation. The main content of the module was previously presented as didactic lectures using power point slides and discussions between the lecturer and students. This is an eight-credit module, on a continuous assessment model, comprising of a test and an assignment.

In an effort to expand and explore alternatives of teaching, photovoice was introduced in 2019. The class of 56 students were introduced to photovoice, as an innovative modality of deepening their engagement with the community by locating themselves in their local societies and capturing photographs of environmental factors that were involved in causing disease. The diverse student cohort are from different parts of the country, many of whom live in student residences. The assignment was opportunistic as it was provided to them prior to a three-week vacation period, to facilitate their planning. In addition, this exercise afforded a diversity of environments, that could be assessed, in various communities.

### Guidelines

Students were requested to work in self- selected groups of two to four per group. Each member of the group was required to capture the photographs of the environment either individually or collectively. They were not restricted in the number of photographs and could use any device inclusive of their cellular phones, cameras or tablets. They were also expected to keep field notes. The students viewed each photograph as a draft on which they made notes. Each group had to subsequently engage in a dialogue, about their photographs. Thereafter, the group consensually chose one photograph for presentation in class.

### Format of presentation

The following questions, associated with the letters in *PHOTO*, which were adapted from Capous-Desyllas and Bromfield [27] guided their presentations.

- Describe the *P*icture.
- What is *H*appening in the picture and how does it affect the *H*ealth of people?
- Why did you take a picture *O*f this?
- What does the picture *T*ell us about the health of people living in the area?
- Can this picture provide *O*pportunities to improve the health of those in the area?

Each group was allocated 10–15 min for the presentation, with a further 5–10 min to respond to questions by other members of the class, the lecturer and an independent assessor, who was not involved in teaching the class.

### Assessment

Each aspect of the project was critically reviewed, so that every part could be assessed. The assessment criteria were guided by the university policy on assessment [28] and the work of Moon [29]. The assessment criteria for the assignment are indicated in Table 1.

**Table 1:** Assessment criteria

Criterion	Mark
**Picture**	
Description	10
Reasons for taking that picture	10
Explanation of how the environmental factors shown affects health of people living in the area	10
An explanation of the diseases that would be prevalent in the area due to the environmental factors as evident from the picture	10
Opportunities to improve health in the area	10
Picture clarity	10
**Presentation Quality**	
Confidence, audibility and maintenance of eye contact	10
**Ability to answer questions**	10

Each of the criteria were assessed on a scale of 0–10, as follows:

- 0–3 – No explanation provided or completely incorrect explanation.
- 4 – Insufficient / details provided.
- 5 – Evidence of an explanation was provided, but it was mediocre.
- 6 – Considerable understanding was demonstrated.
- 7 – A good explanation that was coherent was provided.
- 8 – The explanation was excellent and well explained.
- 9 – The explanation was excellent, showing great command and understanding.
- 10 - A full, comprehensive explanation, with exceptional understanding was provided.

Two academics scored each presentation independently and the average score was used. No major discrepancies were noted between the scoring of both academics. The marks were totaled and converted to a percentage.

### Extending learning into Research

Four groups of students; one from the top, two from the middle and one from the bottom end of the class (as per the marks obtained for the presentation) were purposefully chosen to gauge whether the experience of students with diverse academic capabilities differed. These groups were invited for subsequent focus group discussions regarding their learning experience, from the project. These comprised of a total of 12 students. As data saturation was reached at this point, no further interviews were conducted. Each focus group discussion was conducted for approximately 30–45 min. The focus group discussions were held in a closed room to ensure privacy. The discussions were facilitated by the lecturer as well as an independent researcher, who was not involved in teaching the module. The following questions were used to guide the focus group discussions:

- What technology did you use to obtain the pictures?
- Was it easy / difficult to obtain the pictures and decide what to use?
- Describe any struggles your team had in obtaining the pictures and how you solved this.
- Was it easy to get the required information? (Regarding the picture and the theory related to it)
- Were there any barriers that you faced?
- What approaches did your team use to complete your assignment?
- What factors helped your team feel successful?
- How did you feel about presenting your assignment to the class?
- What was your general attitude towards this type of study?
- What were the benefits of gathering information yourself, as opposed to getting it from the lecturer?

Written informed consent was obtained from all the participants. Permission was also sought to audio-record the focus group discussion for transcription. The data was analysed using Tsech’s eight steps of thematic analysis [30].

 Ethical clearance was obtained from the institutional research ethics committee prior to implementing the project (IREC 108/19).

### Reflexivity statement

The author lectures in Epidemiology: Public Health. Her research interests are in Public Health as well as in Health Science Education.

## Results

The final scores for the group projects ranged between 68 and 94 %, with a mean of 76.8 ± 7.6 %.

The following five themes emanated from the transcripts: affirming the positive student experience with photo-voice, nuanced perceptions of lived realities on community health, inculcating reflective learners, indoctrination of critical thinking and capitalising on unintended community engagement. The words of the students are identified by quotation marks.

### Affirming the positive student experience with photo‐voice

There was a positive and affirming response to cell phone camera usage and none of the participants reported technological challenges as evidenced by: “We were comfortable using our cell phones, we use them for ‘selfies’, anyway” [P8]. This positive experience and comfort with extending cell phone usage for education, was appreciated as facilitating learning away from the classroom. Only one student reported that his original picture was not very clear, hence employing an alternative of a digital camera. “My first attempt - I used this phone but the picture was not that clear. I wanted to keep the quality and then I went for a digital camera” [P5]. This indicates a commitment towards good quality and exploring alternatives for improvement.

The consensual process on picture choice was also reflected upon: “We took many pictures. We looked at each and every picture and then decided that this one was very personal. It shows many issues, including medical issues, so it was very personal to us” [P9]. Another student re-iterated: “We shared ideas to hear what’s the best idea” [P1]. Being afforded this choice was empowering to the students. This dialogue within the group challenged the students to produce the best work. This is indicative of their abilities as co-creators of knowledge as they extend learning beyond the lecture room into the community. The health concerns were identified and framed by the theoretical aspects of their curriculum. Oden [18], supports that allowing the students to seek knowledge, brings about positive relational change.

### Nuanced perceptions of lived realities on community health

The students remarked on individual critical judgement on what specifically depicted environmental factors that potentially lead to ill health “We made sure to observe the whole area as well, because that’s what the presentation was all about. We found many problems about the place and then we decided to go for that one” [P9].

The discernment discussed by P9 suggests their acknowledgement of their lived realities within the community that cause disease, which may not necessarily be contained in their theoretical curriculum. Moreover, it afforded scrutiny about their own actions and their contributions to the interactional situation of cause and effect, especially with disease. In addition, it demonstrated how learning can occur beyond the lecture room and extend into their own communities. Such a relationship with their own environment affords greater applicability to the theory that they learn and allows them to witness theory articulate into practice. “This assignment made us think that small things we do on a regular basis, like dumping all the things, can cause serious disease” [P4]. Evidently, the exercise afforded self-reflection on their liability in contributing to environmental health hazards.

**Fig. 1:**
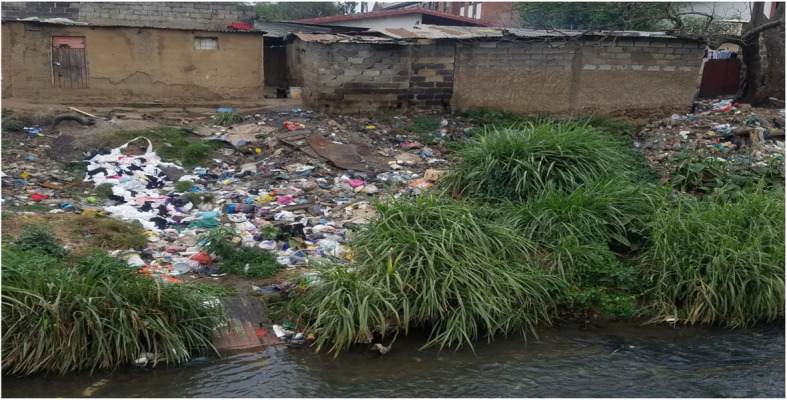
Living conditions in a semi-urban area

Relatedly, one aspect of land pollution was chosen that revealed innumerable interrelated health hazards (Fig. 1). This photograph created the observation of sharp containers in the garbage that elicited a response that: “children playing in the area will get cut” which invariably aligned to related theory on “the cuts will allow filiariform larvae of some nematode parasites, such as *Strongyloides*, which may be present in soil to enter into the body and cause an infection” [P3]. Parasitic worm infections are common in rural areas of South Africa, particularly in areas of poor sanitation, as most infections occur via the feco-oral route [31]. This observation that certain parasites have other modes of entry into the body, which could be exacerbated by litter, indicates contextual thinking, which will promote their development as future professionals and responsive solution focused citizens.

The observation of streams in the vicinity also led them to interrogate downstream movement of waste and its subsequent deleterious effects on other communities. The damp on the walls elicited a discussion of cold damp environments being conducive to pathogens thriving in the area and hence an increased burden of respiratory tract illness. The close proximity of dwellings would lead to an easy spread of communicable diseases. The roof material could lead to asbestosis; “In most cases people think that you have lung cancer only because you smoke cigarettes” [P4]. Students reflected on a variety of causes for a particular disease, with emphasis on local environmental and the concomitant social political context. This is an essential aspect in the current context of decolonisation of the curriculum, which speaks to localizing knowledge within the South African context.

These environmental conditions were also linked to poor local government management. “If you listen to radio, people from these areas are always complaining that the environment around there hasn’t had proper waste removal for like - I don’t know - the past 3 years due to striking. I was happy to hear about it, maybe some other people will try to get involved in what is happening, which is awesome” [P8]. Poor management is predominant in impoverished communities. This can exacerbate the spread of communicable diseases since insect vectors and rodents thrive in areas where garbage is not removed for protracted periods.

**Fig. 2:**
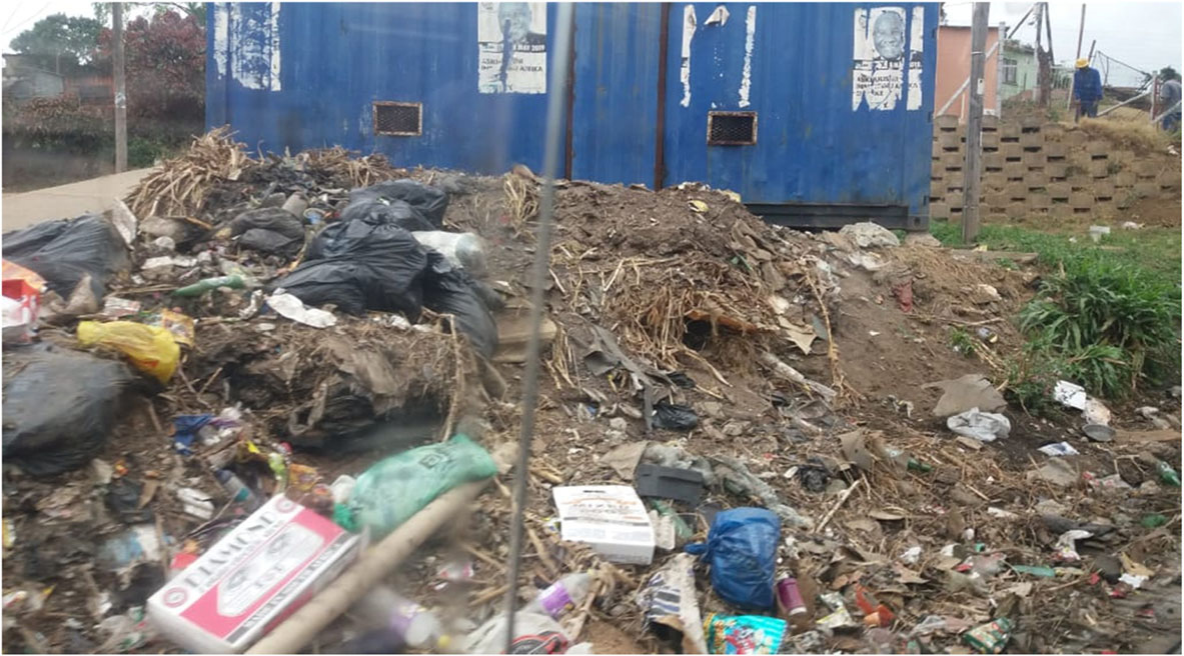
An area behind a clinic, demonstrating medical waste contained in the garbage

Figure 2, is similar except that it is captured from the back of a clinic, with medical waste observed among the garbage. “One of the major reasons why we chose that picture, is the poorly disposed medical waste. Most people weren’t even aware that in some communities there are such problems and making people aware of that was nice, since we do Chiropractic and it’s a medical thing” [P9]. This health hazard of medical waste disposal is a challenge. Although legislations for the proper disposal of medical waste exist, these are not always adhered to. In addition, the effects on children are concerning. Children requiring treatment after eating poisonous tablets and some needing antiretroviral drugs after being pricked with contaminated sharps have been reported [32]. In addition to HIV infection, the latter could also result in Hepatitis B.

It emanated that most pictures captured were from their surrounding community, although not necessarily in the immediate neighborhood. “It’s the surrounding area. It’s a different section from where I live” [P8]. The project thus afforded student engagement beyond their immediate environment as they contemplated surrounding areas that may invariably influence their lived realties within the community.

### Inculcating reflective learners

Evidently, the students were capable of analyzing specific health aspects of the community as captured by their lenses. In particular, they focused on the developmental capacity of such an exercise that facilitated professional growth. “I strongly believe that it does help to build us as professionals; not only how to connect with the work and how to present it to people. You as a professional need to stand in your field and share your views with others and as health practitioners, you need to project confidence and I feel that this presentation has helped with that” [P1]. This exercise promoted incremental confidence as they reiterated the benefits of such an engagement. It instilled an engagement, both with students and the environment, and this sustained space improved their communication skills. It is within this shared learning and teaching environment that learning from each other became pivotal.

There was consensus that engagement in the project afforded appreciative independent thinking that facilitated learning beyond the lecture room; “This assignment was personalized, we had to choose a place. It’s not like something you can find on Google. We had to come up with our own ideas and share that with each other and then compare” [P1]. This reasserted their collaboration as co-creators of knowledge. Another commented that such an exercise allowed them to focus on solutions within the lived health realities confronted by their communities; “When we came to the solution part, you will never ever find that on Google. The solution had to come from us and we had to think how we will solve the problem. How can we come up with a solution when we look at the picture, how can we move this thing from here?” [P2]. Clearly, P1 and P2 reflected on Google as a search engine for solutions, but their experience was rooted in reflection of the photos that incited a solution focused perspective. This demonstrated progression from passive learners to co-creators of knowledge, which certainly recommits to the transformational agenda to ensure lifelong and reflective learners.

### Indoctrination of critical thinking

Knowledge increased as thinking occurred from a new perspective and the understanding was enhanced when other perspectives were added by their classmates. “You grow with knowledge and are able to think outside the box and then when you talk about a certain issue in class, someone adds something else which now makes me richer in terms of knowledge” [P8]. These discussions allowed them to critically interpret the situation so that judgements could be made. “I was brainstorming and I remember the order of everything” [P9]. Another student re-iterated that remembering was easier because they had sought the knowledge themselves “It’s not easy to forget something that you discovered yourself, going through it and come up with the solution and seeing the impact it can cause” [P4]. Moreover, placing the students in an unfamiliar context where they had to decipher the problems enabled them to think unconventionally to advocate innovative solutions. “We knew that we had to have problems and solutions at the end. It made us think deeper and that was an advantage” [P2]. Another student emphasized having the information but not being able to critically analyze a situation would be futile; “Personal experience comes into play with this aspect a lot because you can have the knowledge and be ignorant” [P6]. Clear and rational thinking was thus required to solve the problems that they were faced with. The students demonstrated capability by applying learnt skills in a new context, and to have powers of judgement to discern what was actually happening in the environment. This theme was summed up by P3 “It required me to think critically rather than just answering questions. I think it’s a great way to learn”.

### Capitilizing on unintended community engagement

Some students took the initiative to interview members of the community about the environmental issues that they confronted. The reactions of the community varied. Age was one factor, as younger community members, in contrast to the older generation were both aware and conscientized of the repercussions; “I would say the welcoming people were young people. People in school understand such things but the older people didn’t care at all” [P8]. Another inadvertent aspect was the benefit of extending the teaching environment to include themselves as they occupied the role of a lecturer. Moreover, that space promoted awareness of differing community health challenges which should ultimately invite change; “You can teach other students so that they can notice and change. You can minimize many diseases by doing this” [P4].

It became evident that the photovoice assignment also generated contemplation of preventative measures that were actionable by joining environmental awareness campaigns and participating in recycling projects; “We joined the Litterboom project, which collects plastic waste from rivers. The plastic is then recycled” [P6]. Furthermore, this group encouraged other students to join the recycling project by providing them with its weblink. Such activism, awareness and networking endorsed students as agents of change for a better environment.

Clearly, there was an overwhelming positive response to the photovoice assignment as authenticated by the following participant comments. “I liked it actually. I felt it was more community involved and for the first time in university that I actually had an assignment based on being part of the community, where we had to be there. We had assignments where we spoke about community issues but we didn’t have to be there. This time we had to actually physically take pictures ourselves and see how people react and this really helped with the learning” [P8]. Their presence with their cameras within the community facilitated them to envisage problems within a much broader context. It emanated that such an engagement afforded the reflection of poor local and national governance structures that emphasized adverse environmental conditions.

## Discussion

The used of photovoice as a teaching modality for undergraduate health science students in a module in Public Health revealed an enthusiastically positive response to the incorporation of such an innovative facilitative mechanism within the teaching and learning space. The students demonstrated incremental development as learners who assumed ownership of their learning and occupied the space as co-creators of knowledge. In addition, the multiplicity of benefits had ramifications not only for themselves but, fellow students and the broader community.

The assignment made it feasible for them to record and interpret a large quantity of data. In a similar study, geography students who participated in a photovoice assignment, also supported this contention [19]. Without incurring further financial costs, the use of the cellphone became an extension for learning. Cellphones have been used to augment learning in health sciences [33]. This tool that students are most comfortable with in connecting, demonstrated yet another space of connection within their modules. Its uses within a resource constrained space, where limited finances do not always permit the purchase of expensive teaching equipment, is appropriate. This relates to DUT’s Envision 2030 perspective of sustainability as well as concomitantly providing a distinctive education that is relevant for students [26].

Within the critical gaze of environmental factors that affect health, the students demonstrated how this modality of photovoice facilitated the linking of the social and structural determinants of health within a broader context. Young people identify the social determinants of health that are most important to them, demonstrating that youth may have a bias towards factors that affect their health [23]. South African youth acknowledged over-crowded buildings, inadequate sanitation, substance abuse and reproductive health as their main health challenges [24]. This study also resonates with another study conducted in Cato manor, Durban where the participants indicated a lack of municipal facilities in the area, which they identified as a major health issue faced by their communities [34]. Such an exercise beyond acquainting them with structural inequalities that exist in the community also emphasized the need for municipal intervention to improve the provision of services to marginalized communities. Their awareness was transmitted to the lecture room to stimulate further conscientization of the disparities of public healthcare that exist in diverse communities. Clearly, they interacted with the realities of improving environments to meet the complexity of healthcare demands which require a collaborative approach [12]. It is within such intersectoral collaborative reflections that they realized that a lack of a coordinated agenda in meeting the health risks continue to prevail within their communities. Such realistic interrogations situated for them how the theory of collaboration is valid within the communities and should not be empty rhetoric, but actionable.

Learning was certainly enhanced when compared to studying through didactic teaching. Students demonstrated the ability towards being transformed from passive recipients to active learners. It “stimulated creativity and innovation to generate new knowledge” as required by DUT’s Envision 2030 plan [26]. The exercise afforded moving the learning space beyond the lecture room to the students’ own living contexts or other surrounding environments to gather their data. It demonstrated how reflection is extended to be more engaged as they consider theory and its implications for a broader context. They became acquainted within Public Health as they engaged in exploring health knowledge contextually. The knowledge that they acquired demonstrated how theory is reflective within their lived realties. Moreover, it emphasized the relevance of their community health education. The engagement ensured the development of analytical thinking as they scrutinized their surroundings contextually in order to gauge the multiple ramifications on the health of residents in the area. Critical thinking occurred in response to the task that the students were given and to the problematic situation that they were faced with. In order to deal with these challenges in a critical way, the students had to utilize knowledge gathered in theory whilst applying quality thinking and judgment which according to Bailin [3] are “invaluable tools for critical analysis and evaluation in science”. They were challenged to conduct an enquiry, interpret the observations, and eventually engage in a creative task. The students displayed confidence in working in an unfamiliar context with unfamiliar problems. They were capable of providing reasons for the situation that they were faced with and were able to communicate effectively about that, hence displaying important attributes of a ‘capable education’ [11]. Reflective learners ultimately have an improved awareness of the social determinants of health [35, 36]. Poverty and a low quality of accommodation, particularly in informal settlements have been recognized as important social determinants of health in South Africa [6]. The photovoice assignment enabled the students to ascertain these social determinants through their own critical lenses, to judge how this affected health of the people in the area and to put forward possible solutions. Focusing on community health demands, facilitated the acquisition of competencies through co-creation of knowledge rather than merely through lectures. In particular, the student’s efforts were incorporated into presentations, that not only allocated a mark but offered peer learning, hence empowering them further as key actors in their own learning. It reiterated their communication skills, and confidence improved substantially.

In addition, the students became proactive; they assumed activism roles by joining organizations to clean the environment, to recycle waste and to educate their communities. They became agents for social change as the academic exercise afforded them the opportunity to locate themselves as advocates for such change, becoming responsive to the needs of local communities. They took steps to transform the environment in order to improve the health of the population. This aspect impacted on DUT’s Envision 2030 perspective of establishing “mutually beneficial partnerships”. It “leveraged new knowledge and solutions for societal impact” whilst developing “graduates with the acumen to initiate change” [26]. Students are transformed into innovators for health promotion, as the acquired skills are lifelong [35]. Such incremental benefits facilitate foresight in their journey as lifelong reflective learners.

## Conclusions

Photovoice is an effective method of teaching public health in an undergraduate curriculum. The students affirmed it as a positive learning experience, which enhanced their knowledge and skills. The incorporation of photovoice promoted higher order learning such as problem solving, reflective and critical-thinking. Students through their lens demonstrated evidence of transformation towards critical thinkers who reflected upon theory and how that articulated into practice. Such a dichotomy has been a constant challenge in Higher Education on relevance in the lived realities in the community. This modality has improved and created opportunity for further skills development especially in communication as it enabled them to disseminate knowledge to others. This study has established that teaching using this alternative pedagogy has the potential to produce reflective graduates who are responsive to the existing local health needs of the community. It has established the innovation of the curriculum whilst providing a distinctive education to students who engaged with society. This photovoice project demonstrated the commitment to DUT’s Envision 2030.

## Limitations

This research was conducted on one group of students in the second year of study. Future work should include students enrolled for other programmes as well as those in other years of study to obtain a more comprehensive understanding of the value of the teaching and learning modality across various disciplines.

## Data Availability

This is available upon reasonable request from the author.
